# Economic burden of cardiovascular diseases before and after Iran’s health transformation plan: evidence from a referral hospital of Iran

**DOI:** 10.1186/s12962-020-00250-8

**Published:** 2021-01-03

**Authors:** Vahid Alipour, Hamed Zandian, Vahid Yazdi-Feyzabadi, Leili Avesta, Telma Zahirian Moghadam

**Affiliations:** 1 Health Management and Economics Research Center, Iran University of Medical Sciences, Tehran, Iran; 2 Social Determinants of Health Research Center, Ardabil University of Medical Sciences, Ardabil, Iran; 3 Department of Community Medicine, School of Medicine, Ardabil University of Medical Sciences, Ardabil, Iran; 4 Health Services Management Research Center, Institute for Futures Studies in Health, Kerman University of Medical Sciences, Kerman, Iran; 5 Department of Cardiology, Ardabil University of Medical Sciences, Ardabil, Iran

**Keywords:** Cardiovascular disease, Economic burden, Health transformation plan, Health system reform, Referral hospital

## Abstract

**Background:**

Different countries have set different policies to control and decrease the costs of cardiovascular diseases (CVDs). Iran was aiming to reduce the economic burden of different disease by a recent reform from named as health transformation plan (HTP). This study aimed to examine the economic burden of CVDs before and after of HTP.

**Methods:**

This cross-sectional study was conducted on 600 patients with CVDs, who were randomly selected from a specialized cardiovascular hospital in the north-west of Iran. Direct and indirect costs of CVDs were calculated using the cost of illness and human capital approaches. Data were collected using a researcher-made checklist obtained from several sources including structured interviews, the Statistical Center of Iran, Iran’s Ministry of Cooperatives, Labor, and Social Welfare, the central bank of Iran, and the data of global burden of disease obtained from the Institute for Health Metrics and Evaluation to estimate direct and mortality costs. All costs were calculated in Iranian Rials (IRR).

**Results:**

Total costs of CVDs were about 5571 and 6700 billion IRR before and after the HTP, respectively. More than 62% of the total costs of CVDs accounted for premature death before (64.89%) and after (62.01%) the HTP. The total hospitalization costs of CVDs was significantly increased after the HTP (p = 0.038). In both times, surgical services and visiting had the highest and lowest share of hospitalization costs, respectively. The OOP expenditure decreased significantly and reached from 54.2 to 36.7%. All hospitalization costs, except patients’ OOP expenditure, were significantly increased after the HTP about 1.3 times. Direct non-medical costs reached from 2.4 to 3.3 billion before and after the HTP, respectively.

**Conclusion:**

Economic burden of CVDs increased in the north-west of Iran after the HTP due to the increase of all direct and indirect costs, except the OOP expenditure. Non-allocation of defined resources, which coincided with the international and national political and economic challenges in Iran, led to unsustainable resources of the HTP. So, no results of this study can be attributed solely to the HTP. Therefore, more detailed studies should be carried out on the reasons for the significant increase in CVDs costs in the region.

## Background

Cardiovascular diseases (CVDs) are among the leading causes of death and the essential reason for premature death (before 65 years old). In 2017, CVDs were responsible for 17.7 million death around the world, which is around 31.8% of the total deaths in the world [1]. Cardiovascular diseases are the cause of 161,000 deaths in Iran, which accounted for 45.45% of all deaths in the country in 2016. The age of CVDs has dropped and the incidence of CVDs among the population under 70 years has steepened during the last decade and reaching from 569 in 2017 to 484 per 100,000 people [1, 2]. This trend for Europe had been estimated to be about 700 deaths among people under the age of 65 years in 2017 [3]. In Iran, this trend has also been tangible, and the rate of CVDs has been increased before the age of 60 years [4]. Increasing the rate of CVDs is one of the factors contributing to the increasing costs and economic burden of the disease [5]. Ardabil, one of the north-west provinces of Iran, has the highest rate of CVDs incidence and prevalence around the country. The proportion of deaths from CVDs was 2319 in 2015, accounting for 48.46% of all deaths in the province, which is higher than the world’s and Iran’s averages. The mortality rate of the disease in Ardabil had been estimated to be 180 cases per 100,000 people in 2017 [6, 7].

Cardiovascular diseases costs are very high, so that in the European Union (EU) in 2016, the direct and indirect costs (premature death and disability) of CVDs were estimated to be about 25 and 18 billion euros, respectively [3, 8]. It has also increased the inequality in health care financing by increasing OOP expenditure and catastrophic health expenditures [9]. In the US, the direct and indirect cost of CVDs was about 329.9 billion USD, and it is estimated to increase to 1.1 trillion USD in 2035 [10]. The economic burden of CVDs in developing countries, such as Iran, which accounts for more than 75% of death rate, is more sever [11]. The economic burden of CVDs could have adverse effects on the country productivity and competitiveness, increase fiscal pressure on the health systems, and increase poverty, inequity, and opportunity loss in developing countries [12].

Different countries, especially low-income and developing countries, have implemented several reforms in their health system framework to prepare financial protection to patients by decreasing the economic burden of diseases [13, 14]. To this end, one of the most recent reforms in the Iranian health system, called the Health Transform Plan (HTP) with eight interventions for increasing equity in the health system, was implemented from early 2014 [15]. The central intervention of the HTP was reducing OOP expenditure for inpatient services in the hospitals affiliated with the Ministry of Health (MoHME) [15–17]. It also contains other interventions, such as encouraging physicians to stay in deprived areas and improving quality of care in the hospitals affiliated with MoHME through different interventions, such as increasing specialists, improving quality of outpatient services of the attached polyclinics, and improving hospital amenities and lodging services [15, 18, 19]. These interventions have had different effects on patients’ satisfaction and equity in the health system financing [19–22]. The first intervention of the HTP aimed to decrease patients OOP expenditures as direct costs of the disease, and other interventions aimed to reduce indirect costs of disease through increasing access to health care services [18, 21].

Therefore, due to the high rate of CVDs in Iran, especially in Ardabil, as well as the implementation of the HTP as the essential equity-based intervention of the health system, this study was conducted to estimate the economic burden of CVDs before and after the HTP in the north-west of Iran.

## Methods

This cross-sectional study was conducted during September 2019 to February 2020, to evaluate the economic burden of CVDs in two periods: before (2013) and after (2016) the HTP implementation. The bottom-up approach, the cost of disease, and the human capital approach (HCA) were used to calculate the total costs.

### Study design and setting

This cross-sectional study was conducted before and after the HTP implementation in the north-west of Iran. A 550-bed cardiovascular specialized hospital affiliated with MoHME was selected as the study site. At both time points (before and after the HTP), data on direct, indirect, and non-medical costs were collected from hospital-based cardiovascular inpatients records and compared. Baseline data were collected from households with CVDs at two intervals.

### Eligibility criteria

Patients with one of the CVDs (coronary artery stenosis, heart failure, heart attack, and cardiac rheumatism) in two periods, before (March 2012 to March 2013) and after (March 2016 to March 2017) the HTP, were eligible to participate in the study. Given that most hospitalization costs occur in the early years of the diagnosis and treatment of CVDs, the diagnosis of the disease over the past year was the basis of inclusion.

### Sampling methodology and sample size

Samples were collected from a specialized cardiovascular hospital the most extensive and only reference for patients with CVDs using a purposive random sampling method. Initially, a list of all patients admitted to the study during the two intervals was prepared, and data were collected from inpatients medical records through a checklist. If necessary, patients who were eligible to participate in the study were contacted, and additional information was obtained via telephone interview.

Four types of CVDs with high frequency (coronary artery stenosis, heart failure, heart attack, and cardiac rheumatism) were selected to estimate sample size. Then, the medical records of 40 discharged patients were randomly selected and evaluated for cost. Based on the mean and variance of hospital costs and using Cohen’s sampling method, the sample size was estimated to be 253 (n = 800) before and 284 (n = 1300) after the HTP.

Cohen’s *d* is simply a measure of the distance between two means, measured in standard deviations [23]. It is calculated using Eq. (1):

1 $$d=\frac{{M}_{1}-{M}_{2}}{{SD}_{pooled}}$$

where *M*_*1*_ and *M*_*2*_ are the means for the 1st and 2nd samples, and *SD*_*pooled*_ is the pooled standard deviation for the samples. *SD*_*pooled*_ is appropriately calculated using the following equation:

2 $${SD}_{pooled}=\sqrt{\frac{\sum {({X}_{1}-{\stackrel{-}{X}}_{1})}^{2}+\sum {({X}_{2}-{\stackrel{-}{X}}_{2})}^{2}}{{n}_{1}+{n}_{2}-2}}.$$

Accordingly, the total number of hospitalizations for CVDs was extracted from the hospital information system (HIS), then, 600 patients for both intervals (300 before and 300 after the HTP) were randomly selected using random number table.

A pilot study was conducted a month before (August 2019) on 30 inpatients with CVDs by a face-to-face interview to estimate indirect costs in the last month. The data were extracted using a checklist. Data were multiplied by 12 to estimate the total costs in the last year. Based on the mean and variance of hospital costs and using Cohen’s sampling method, the sample size was estimated to be 180 for both time points, before and after the HTP implementation. By telephone follow-up, patients were selected from the direct costs calculation phase (n = 300). Contact with all selected patients continued until the designated sample size was obtained.

### Data collection

Data were collected using a researcher-made checklist included age, sex, length of hospital stay, hospitalization costs, and other related costs (visitation, diagnostic services, hoteling expense (bed-days expense), surgery, and medication), which were extracted from the patients’ medical records. A checklist used in other similar international studies was used to collect non-hospital and non-medical costs [24, 25]. The questionnaire consisted of two sections; the first section included demographic information (age, gender, marital status, education status, and the number of family members), duration of illness, and insurance information. Also, the second section included information on indirect costs (absenteeism, sick leave, disability, etc.).

### Study variables and data sources

In this study, multiple data sources were used to estimate the economic burden of the disease. The most important data source used to estimate direct medical costs were the information extracted from the medical records of the patients with CVDs.

#### Direct costs

This type of costs is directly attributable to patients admitted to various types of health care facilities (Ministry of Health, Social Security, and private sector), as well as treatment costs, including different services, such as diagnostic, medical, and rehabilitation services [26]. To estimate direct costs, the costs of standard diagnostic services, such as electrocardiograms, echocardiography, exercise testing, nuclear heart scan, and angiography, were included. Sampling was performed among patients who had undergone open-heart surgery, angioplasty, and medication. Sample sizes for the three groups (undergone open-heart surgery, angioplasty, and medication) were 135, 251, and 221 people, respectively. Sampling was performed using simple random sampling method and Cochrane-Orcutt Procedure, which is applicable for a limited population, was used. The main drugs for CVDs were also extracted by reviewing 65 patients’ medical records and interviews with CVDs specialists. In this section, direct payments were considered as the patients’ OOP expenditure, which can be due to the difference between the patient’s share of the cost and share of the insurance cost, which was recorded as the patient’s share of the costs in their medical record.

#### Indirect costs

Indirect costs are a combination of the costs incurred to the patients and their families, such as the cost of medical travel, cost of time lost, and cost of patient in-home care [27].

#### Time costs

Time costs are calculated based on the mean number of days lost due to receiving health care services and hospitalization in the hospital. In this study, time costs were calculated by multiplying the number of hospitalization days by the average daily wage. Data for an average daily wage were obtained from the report of the Ministry of Cooperatives, Labor, and Social Welfare of Iran in 2018.

#### Premature death costs

The human capital approach was used as the primary method for the estimation of premature death costs in this study. In previous studies, HCA was used for the estimation of premature death due to non-communicable diseases, such as CVDs [28, 29]. Total numbers of deaths due to CVDs during last 5 years were obtained from documents of the hospital and deputy of the health of ARUMS. The data adopted to the data of the Institute for Health Metrics and Evaluation (IHME) and Global Burden of Disease (GBD), and then, registered.

### Data sources and manipulation

Data were collected using structured interviews with patients, medical records, and valid data sources.

- Population, employment rate, and life expectancy were extracted from the latest report provided by the Iranian Statistic Center (ISC).
- CVDs morbidity and mortality data were extracted from the National and Subnational Burden of Diseases, Injuries, and Risk Factors (NASBAD) survey.
- The final report by the Central Bank of the Islamic Republic of Iran was used to identify and determine the exchange rate by year.
- The average daily cost (the daily minimum wage lost) was calculated according to the principles presented by the Ministry of Cooperatives, Labor, and Social Welfare of Iran.
- The difference between payments by patients (patient’s share) and the insurance liability was calculated to determine the OOP expenditure.
- Indirect costs identified by data extracting through interviews with patients and their relatives who were aware of patients’ diseases using the designed checklist.
- Given that, there were no precise statistics on the level of household income, their income was estimated based on the minimum declared incomes of the Ministry of Labor, Cooperatives and Social Welfare.
- Other information, such as disease incidence, occupancy rate, payroll, GDP, national health expenditures, and standardized life expectancy were extracted from previous studies, treatment guidelines, World Bank databases, Iranian Ministry of Health (MoHME), and Iranian Statistic Center (ISC).
- All costs were equalled and presented in Iranian Rials (IRR) in two periods (the average exchange rate in 2013 equals 1$ = 31,840 IRR and in 2016 equals 1$ = 36,429 IRR).

### Data analysis

The human capital approach considers the valuation of waste resulting from premature death for the whole economy [30]. Individuals play an economic role in society by generating, earning, and consuming. Thus, by calculating production, income, or consumption, the extent of the impact of one’s economic activities can be estimated.

In the present study, the discount rate of 3% was used to obtain the present value of the lost production. The Present Discounted Value of Lifetime Earnings (PVLE) model developed by Max et al. (2000) was also used to calculate and estimate the present value of the patients’ income [31].

Data were analyzed using SPSS version 16 (SPSS Inc., Chicago, IL, USA) [32] and Excel software version 2013. Continuous variables are reported as means ± standard deviation. Categorical variables are reported as proportions (%). Continuous variables were compared using t‑test and one‑way ANOVA, and categorical variables were compared using the Chi‑square test.

## Results

The prevalence of CVDs in Ardabil before and after the HTP was 44.12% and 50.11%, respectively. The number of deaths due to CVDs in Ardabil before and after the HTP was 2627 and 2940, respectively that accounts for 42.3% and 45.4% of the total deaths in the province, respectively.

### Demographic status

The results showed that 68.8% and 65.3% of the participants were men before and after the HTP, respectively. The mean age of patients in the two intervals was 62.4 ± 9.2 and 62.6 ± 8.6 years, respectively. The minimum and maximum hospital stay were one to 11 days, while the mean length of hospitalization in the two periods (before and after the HTP) was 1.5 ± 1.3 and 1.5 ± 3.9 days, respectively (Table 1).

**Table 1:** Baseline demographic characteristics of patients with CVD before and after HTP in north-west of Iran

Demographic characteristics	Before HTP	After HTP	P-value
Total subjects	300	300	
Age mean (years)	62.43	62.61	0.881
Age groups, %			0.865
< 35	3.73	3.84	
35–44	6.08	6.07	
45–54	13.48	13.49	
55–64	26.34	26.33	
65 +	50.37	50.27	
Gender			0.224
Male	68.6	65.3	
Average length of stay (ALOS)	3.42	3.88	0.702

### Hospitalization costs

Data were extracted from the medical record of patients to estimate the hospitalization costs. The average cost of hospitalization per patient was 2.96 ± 4.18 million IRR, which was 3.69 and 4.64 million IRR before and after the HTP, respectively. The average daily cost of hospitalization before and after the HTP was 1.04 and 1.28 million IRR, respectively, and the total daily cost of hospitalization was 3.06 ± 1.10 million IRR. The total cost of hospitalization before and after the HTP was 6.51 and 8.47 billion IRR, respectively, indicating that it was increased about 1.3 times more. The distribution of direct hospitalization costs in patients with CVDs by service type is presented in Table 2.

**Table 2:** Average and total cost of hospitalization for patients with cardiovascular disease in Ardabil (North-west of Iran), 2018

Service item	Before HTP			After HTP			Prec. change	P-value
	Mean cost	Total cost	Total cost (%)	Mean cost	Total cost	Total cost (%)		
Diagnostic tests	4,902,546	1,416,345,612	13.3	6,733,684	1,935,260,657	14.5	1.37	0.000
Medication and drugs	6,745,609	2,005,469,481	18.3	8,869,887	2,613,068,600	19.1	1.30	0.000
Surgical services								
Open surgery	14,080,998	1,824,897,276	38.2	15,185,617	2,154,839,112	32.7	1.18	0.000
Angiography/angioplasty	7,814,585	307,113,191	21.2	11,191,846	496,917,984	24.1	1.62	0.000
Visiting	1,142,699	342,809,625	3.1	1,486,054	445,816,291	3.2	1.30	0.000
Hoteling expence^a^	2,248,536	614,524,957	6.1	2,972,109	826,543,404	6.4	1.35	0.000
OOP as a proportion of household total expenditure			54.2			36.7	0.67	0.000

*HTP*  health transformation plan, *OOP*  out of pocket, *Prec.change*  percentage of change

^a^Refers to the inpatient's overhead cost for the bed, patients companion and the cost of food and other non-medical expenses

**Fig. 1:**
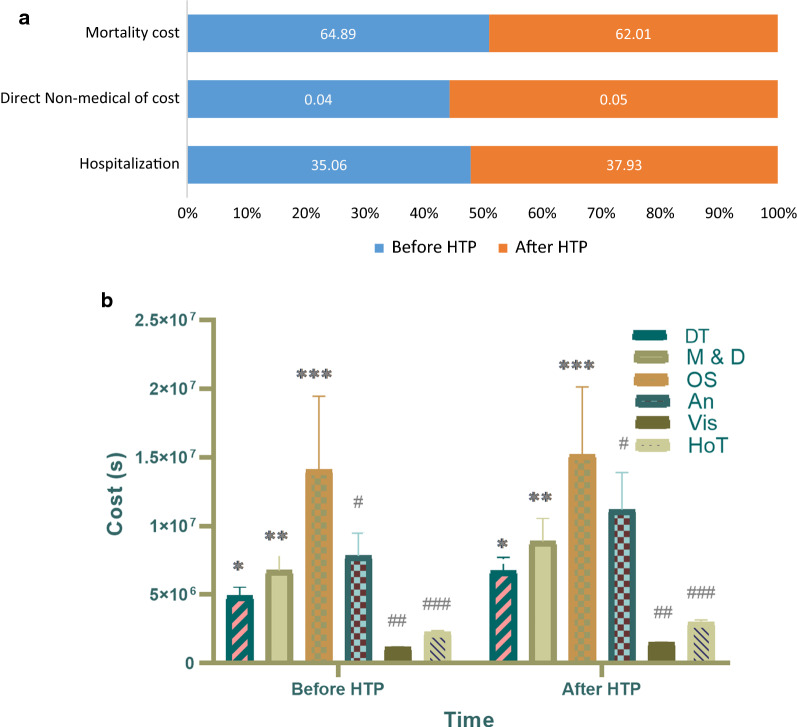
**a** Main component of cardiovascular disease cost in North-West of Iran before and after HTP. **b** Hospitalization costs of cardiovascular diseases (CVDs), before and after of health transformation plan (HTP). Values are presented as mean and standard deviation (SD). Based on estimation there were significant difference between diagnostic tests (DT) *p < 0.001, medication & drugs (M & D) **p < 0.001, open surgery (OS) ***p < 0.001, angiography/angioplasty (An) #p < 0.001, visiting (Vis) ##p < 0.001, and hoteling expense (HoT) ###p < 0.001 before and after of HTP in CVDs

Based on the total number of patients with CVDs identified and the average cost of hospitalization over the two time periods, the total cost of hospitalization of patients with CVDs in the two time periods was estimated to be 1953 and 2541 billion IRR, respectively. Most of the hospitalization costs in both periods were related to surgical services (59.4% and 56.8%) and medication (18.3% and 19.1%). The ratio of indirect costs to the total hospitalization costs was reduced by 33%. In terms of all costs associated with providing services to patients with CVDs, except OOP expenditure, there was an increase in the costs after the HTP, while the highest rate belonged to the cost of open-heart surgery and the lowest one was related to angioplasty and angiography (Fig. 1).

### Non-medical direct costs (lost time costs)

In the present study, the patients’ direct non-medical costs (time and travel costs) were identified and calculated by telephone interviews with the patients before and after the HTP.

Before and after the HTP, 60.6% and 57.1% of the patients were employed (self-employed, government employee, and retired persons) and others were unemployed (unemployed, housewife), respectively. The mean total days lost due to the diseases for both groups of patients (employed and unemployed) were estimated to be 61 days (17 days due to medical travel and 44 days due to disability). The average cost per patient per day was calculated based on the mean income. For this purpose, the average monthly income of employed persons was calculated and divided into 30 days, and one-day income was calculated, which was 0.28 and 0.33 million IRR before and after the HTP. The average daily cost of unemployed patients was also calculated based on the minimum wage, which was declared by the Ministry of Cooperatives, Labor, and Social Welfare in the two time periods. Accordingly, the average daily cost of unemployed patients (minimum wage lost daily) was estimated to be 0.16 and 0.27 million IRR before and after the HTP.

The total cost of time lost due to medical travelling by employed patients was estimated to be 4.80 and 5.63 million IRR before and after the HTP. The total cost of time lost due to disability was estimated to be 12.42 and 14.59 million IRR before and after the HTP. The total cost of travel and disability of employed patients before and after the HTP was 1.87 and 2.08 billion IRR, respectively, and for unemployed patients, it was estimated to be 541.94 and 734.35 million IRR before and after the HTP, respectively. The average cost of time lost due to medical travel of unemployed patients was 2.76 and 4.60 million IRR before and after the HTP, respectively. The cost of time lost due to disability in the two time periods was estimated at 7.14 and 11.91 million IRR, respectively. The total cost of time lost for the unemployed patients was estimated to be 0.526 and 1.27 billion IRR before and after the HTP, respectively.

### Indirect cost (premature death and disability)

Indirect costs were estimated in terms of premature death and mean life expectancy for both sexes (75.15 years in 2013 and 75.95 in 2016). In Ardabil province, 880 people with ages of 35 to 75 years died due to CVDs in 2013, which was reduced to 782 in 2016. The total cost of premature death costs was 3615.3 and 4155.4 billion IRR before and after the HTP, respectively (Table 3).

**Table 3:** Total cost of cardiovascular disease in North-West of Iran (Ardabil), 2013–2016

Service item	Mean cost		Total cost (%)		p-value
	Before HTP	After HTP	Before HTP	After HTP	
Hospitalization	1,953,348,042,488	2,541,733,814,194	35.06	37.93	0.038
Direct non-medical of cost	2,404,333,500	3,355,790,560	0.04	0.05	0.041
Mortality cost	3,615,314,347,567	4,155,499,500,311	64.89	62.01	0.081
Total cost	5,571,066,723,555	6,700,589,105,065	100	100	0.047

### Total economic burden of CVDs

The total cost of CVDs in the north-west of Iran (Ardabil) before and after the HTP was 5571.06 and 6700.58 billion IRR, respectively. Overall, the results showed a significant difference in the hospitalization and indirect costs (medical travel and disability costs) of patients before and after the HTP (P < 0.05). However, despite the relative increase, the indirect and indirect mortality costs were not significantly different (P < 0.05).

## Discussion

Cardiovascular diseases (CVDs) are among costly diseases for patients, their families, and health systems around the world. Different health systems trying to control and decrease the costs of CVDs by different reforms and restructures with a non-communicable disease control approach [33]. Health transformation plan (HTP) is the main reform in Iran’s health system toward the development of health care equity in the country [15, 22]. One of the main objectives of the HTP was providing fair financing and accessibility to the health care services [16, 22], which was expected to reduce the economic burden on Iran’s health system. The prevalence of CVDs in Iran was 179 and 194 per 100,000 people before and after the HTP, respectively [1].

This study aimed to evaluate the economic burden of CVDs in the north-west of Iran, an area with the highest incidence and prevalence of CVDs [5], before and after the HTP to identify the impact of the plan on the CVDs’ economic burden indexes. Based on the results of the present study, the total economic costs of CVDs were estimated 5,571,066,723,555 and 6,700,589,105,065 IR before and after the HTP, respectively. It shows that the total costs of CVDs significantly increased about 1.2 times after the HTP and caused more inequitable status for patients with CVDs in the health system of Iran. This finding is inconsistent with the findings of other studies, where a qualitative study in Iran concluded that the HTP in all dimensions has reduced costs and justified the health system [22]. The finding is inconsistent with the results of a study by Chapman et al. (2011), where they claimed that inpatient costs of CVDs over time were decreased [34]. The difference between the findings of the present study and those of Chapman et al. (2011) may be due to the difference in the context of the studies and methodology.

This study showed that mortality cost is one of the main components of CVDs economic burden in the north-west of Iran in both periods (before and after the HTP) so that more than 62% of the total costs of CVDs can be attributed to the mortality costs. The study also showed that despite a reduction in the percentage of the CVDs mortality cost after the HTP, this reduction was not statistically significant compared to that before the HTP, which is not consistent with the findings of most studies in developed countries. In European countries, more than 60% of the total costs of CVDs attributed to the hospitalization [35, 36], while in other studies, it was reported that just about 23% of CVDs costs attributed to the mortality costs in the UK [24] and about 33% in South Korea [37]. One of the reasons for this may be due to the low mean age of CVDs mortality and morbidity in developing countries, such as Iran [38]. In 2017, the mortality rate of CVDs in Iran and countries with high socio-economic indexes was about 271 and 125 per 100,000 people per year, respectively [1]. A cohort-based study in Iran (2013), showed that CVDs mortality rate in men and women was 331 and 203 per 100,000 people per year, which is almost two times higher than the average income of countries [39]. These pieces of evidence suggest that premature mortality and morbidity increased the proportion of mortality costs compared to the other costs of CVDs in the north-west of Iran. Besides, it is worth mentioning that the costs of health service in Iran are lower than those in developed countries and some other Middle Eastern countries [26, 40], and this can lead to a lower proportion of hospitalization costs to the total CVDs costs in Iran.

Furthermore, it was revealed that surgical services accounted for the highest proportion of costs compared to the other components of hospitalization costs before and after the HTP, which means that the total costs of open surgery and angiography/angioplasty increased more than other hospitalization costs after the HTP. In the UK, hospital inpatient care was estimated about 63% of the total CVDs costs, and surgical services were introduced as the largest share of hospitalization costs [24], which confirms the results of the present study. Another study in Serbia showed that more than 60% of the costs are attributed to hospitalization and surgical services, which is consistent with the results of the present study [36]. Similarly, another study on patients with CVDs in Iran reported direct care costs as a significant proportion of the costs associated with open-heart surgery costs [41].

The proportion of hospitalization costs have been increased significantly after the HTP. One of the leading causes of this increase could be increasing hospitalization rate after the HTP. Karami et al. (2017) reported that the hospitalization rate was increased after the HTP in Iran [42]. Tabari-Khomeiran (2019) also showed that CVDs hospitalization costs increased significantly after the HTP [21]. After the implementation of the HTP, access to health services and responsiveness have been significantly increased, and as a result, health care expenditures in Iran have also been increased [18, 43].

The main objective of the first phase of the HTP was decreasing OOP expenditures of inpatients based on the patients’ insurance type (rural or urban) by allocating subsidies to increase the share of government health expenditures [15, 16, 44, 45]. However, the results of the present study showed that the mean total hospitalization costs after the HTP implementation have increased by 20%. However, OOP expenditures decreased by about 30% after the HTP for CVDs in this study. According to various studies, the share of OOP expenditures has declined by an average of 20% after the HTP, from about 58% to 37% [20, 21]. After the implementation of the HTP in Turkey, patients OOP and their financial share of health system decreased [46]. Accordingly, it can be concluded that despite the decrease in the OOP expenditures and patient’s share of health service payment due to the HTP, the economic burden of CVDs increased. This increase can be due to several reasons, including:

- Increasing hospitalization rate and the average length of stay: the increase of the average length of stay can be due to the increase of patients satisfaction and decrease of personal discharge rate [47].
- Lack of initiatives, such as failure to fully implement the referral system, and increasing referrals to specialized levels of health care system led to system failure to achieve Universal Health Coverage (UHC) [48, 49].
- Lack of timely and appropriate allocation of defined resources after the HTP caused a failure to fully adhere to the HTP guidelines [18, 50].
- The increase of outpatient OOP, where Rad et al. (2017) reported a significant increase of OOP payments for outpatient care services increased after the HTP, which influenced the total OOP [19]. Also, despite the increase in the share of health from GDP after the HTP in Iran, catastrophic and impoverishment expenditures have not decreased sufficiently to achieve the goals of the HTP in term of the financial risk protection [51].
- Failure to fully implement the HTP and its related laws, as Sajadi et al. (2019) reported that unsustainable resource allocation is one of the main challenges of Iran’s health system before and after the HTP [52]. This has led to the failure of most of the formulated policies to achieve their goals on the path to UHC.
- Finally in developing countries, the increase of the costs of health care services with a decrease of patients OOP expenditures can mean the provision of the costs to the public budgets, which can be interpreted as an increase in the government subsidies to the health sector [16]. Therefore, it can be claimed that the Iranian government has increased the health system subsidies by shifting resources from targeting subsidies and one per cent value-added tax to the health system, which is confirmed by similar studies [53, 54].

However, this study had several strengths and limitations related to the study design and costs measurement. First, a province from the north-west of Iran (Ardabil), which has the highest level of CVDs, was selected, and the economic burden of CVDs that has not been studied before, was investigated. In addition, this study, for the first time, investigated the HTP and its effect on the economic burden of CVDs in Iran. In this regard, the evaluation of the economic burden of CVDs before and after the implementation of the main reform in Iran’s health sector, which may help policy makers to reorder the existing policies incorporated in the HTP in terms of affordability of the use of valid databases and robust methods with acceptable sample size to identify and capture the expenditures, is another strength of this study. Classification of the costs into three groups in general, non-calculation of outpatient costs and informal payments, and the possibility of recall bias in the patients during the study period, especially regarding indirect and non-medical costs, and excluding some demographic data because of high rate of missing can be considered as the main limitations of the present study.

## Conclusion

According to the results, after the implementation of the HTP in Iran, the economic burden of CVDs increased significantly (about 1.2 times), especially in the mortality costs and inpatient surgical services costs. Therefore, it can be concluded that the HTP, despite the decline in OOP expenditures and patients’ share of the total health expenditures, the economic burdens on the health system because of several reasons, including failure to fully implement the referral system and increased referrals to the specialized levels of the health system, increased while the OOP expenditures decreased. As a result, the total costs of the health system increased and imposed pressure on the country’s general budget. Finally, based on the HTP objectives in terms of equity in health care financing and the results of this study, it can be concluded that the costs of CVDs in Iran are significantly high. The HTP has not touched its all goals about CVDs costs. It could be because of the non-allocation of financial resources defined to the HTP, one per cent value-added tax, and 10% of targeted subsidies, after the implementation of the plan, as well as the political and economic challenges occurred during the years following the HTP implementation in Iran. Further studies are needed to find ways to modify the challenges and reduce the economic burden of CVDs by the HTP in Iran.

## Data Availability

The data sets used and/or analysed during the current study are de-identified and available from the corresponding author on reasonable request.

## Abbreviations

CVDs: Cardiovascular Disease
HTP: Health transformation plan
IRR: Iranian Rials
OOP: Out of pocket
USD: United States Dollar
MoHME: Ministry of Health and Medical Education
HCA: Human capital approach
IHME: Institute for Health Metrics and Evaluation
GBD: Global burden of disease
ISC: Iranian Statistic Center
NASBAD: National and subnational burden of diseases, injuries, and risk factors
GDP: Gross domestic product
PVLE: Present discounted value of lifetime earnings
